# The Effects of Implementing Gamification in the Hepatology Curriculum for Medical Students

**DOI:** 10.7759/cureus.66538

**Published:** 2024-08-09

**Authors:** Chelsea Edirisuriya, Jason Goldenberg, Zachary Breslin, Anita Wilson, Steven Herrine, Christina Tofani, Danielle Tholey

**Affiliations:** 1 Internal Medicine, Thomas Jefferson University Hospital, Philadelphia, USA; 2 Internal Medicine, Sidney Kimmel Medical College, Thomas Jefferson University, Philadelphia, USA; 3 Psychometrics, Thomas Jefferson University Hospital, Philadelphia, USA; 4 Gastroenterology and Hepatology, Thomas Jefferson University Hospital, Philadelphia, USA; 5 Gastroenterology and Hepatology, Cooper University Hospital, Mount Laurel Township, USA

**Keywords:** medical student, online medical education, medical education curriculum, hepatology, gamification technique

## Abstract

Background

Gamification, the incorporation of game theory into the curriculum, has been correlated with improved knowledge retention compared to standard didactics.

Objective

To determine the impact of gamified hepatology modules on medical student knowledge retention and exam performance.

Methods

We created three web-based, gamified hepatology modules with 15-question pre- and post-tests. Differences in each module’s pre- and post-test scores were compared using paired t-tests.

Medical school exam scores (total score and hepatology-specific score) in module users versus non-users were compared using independent two-sample t-tests.

Results

Module completion yielded significant increases in pre- to post-test scores for the jaundice (p=0.002) and anatomy modules (p<0.001). Module users scored 1.2 points higher on the total exam score (p=0.4) and 2 points higher on the hepatology exam score (p=0.31). Post-module survey results revealed higher knowledge retention in hepatology topics, expanded interest in hepatology, and an increased inclination to use web-based learning platforms for future learning experiences.

Conclusion

Module use led to improved post-test scores in the modules. Module users also had higher hepatology exam and total exam scores, yet, this difference was not statistically significant.

Overall, this study suggests that gamification may be beneficial in aiding hepatology knowledge recall.

## Introduction

Gamification utilizes traditional game elements and applies them to educational activities. The concept’s popularity continues to rise and evolve as students have a high degree of technological literacy and a desire for diverse educational experiences [1]. The era of gamification has proven to be an advantageous way for medical students to learn outside of the lecture or textbook setting. Health profession educators are using gamification to improve students’ learning outcomes through educational games, online modules, mobile medical applications, and virtual patient scenarios [2].

The gamified curriculum provides a myriad of potential benefits in medical education, including the learner’s ability to engage in risk-free healthcare decision-making, remote learning, learning analytics, immediate direct feedback, and improved scaffolding for recall [2,3]. Formats that encourage content retrieval have been shown to enhance learning by forming memories that are more durable in future applications [4]. Systematic reviews of these gamified learning platforms have demonstrated enhanced knowledge retention in medical students [2]. However, most of these studies rely on student perception, and there remains a paucity of data on hard outcomes. Using the gamified Kahoot! platform, Neureiter D et al. demonstrated that interactive histopathology games led to significant improvements in mean pretest versus posttest scores from 47% to 77% [5].

Our study applies gamification to a hepatology curriculum for medical students, which has not yet been studied. Hepatology is a complex subject requiring the learner to master anatomy, histology, biochemical processes, and clinical knowledge. Thus, we query if gamification can help simplify these complex concepts. We aim to assess the effect of online, gamified hepatology modules on medical student knowledge recall and exam performance. We also aim to compare the impact of this learning intervention with the exam performance of medical students who used only the standard didactic methods. Our goal is to assess the value of applying gamification to a hepatology curriculum and gain insight into which game elements are most effective. We hypothesize that the use of a gamified hepatology curriculum will result in increased knowledge recall, enhanced learner engagement, and improved overall and hepatology-specific test scores compared to those who utilized standard didactic measures.

## Materials and methods

Patient population and module design

Our study was conducted at a large academic medical college in Philadelphia with a population of first-year medical students. Module completion and post-module survey data were collected between March and May of 2022. All data collection was deidentified, and study methods were performed in accordance with our center’s institutional review board.

A multidisciplinary team, including medical students, residents, attendings, an education curriculum consultant, and a graphic designer, created three web-based, gamified hepatology modules on liver anatomy, evaluation of jaundice, and interpretation of elevated liver function tests (LFTs) using the Articulate platform (Adam Schwartz, Articulate, Articulate 360, 2002). The modules utilized interactive material including animated videos, educational games, mnemonics, matching exercises, interactive diagrams, simulations, case-based learning, and board style questions (Appendix 1). To test the effectiveness of knowledge recall and the modules’ ability to cover their objectives, we created 15-question pre and post-tests.

All students were provided free access to the modules during their first-year medical college Gastroenterology (GI) course, via our university’s course education website on Canvas (Devlin Daley, Canvas, Instructure Learning Platform, 2008). The first 25 students to complete the modules were incentivized by a $25 gift card. However, student participation was completely voluntary, and participation did not in any way affect students’ course grades. This incentive approach was outlined and approved by the IRB board, and students signed a written consent outlining this process prior to their participation in the study.

Statistical analysis of gamified modules

Differences in each module’s pre- and post-test scores were compared using paired t-tests, with students serving as their own internal controls. We also compared medical school exam scores in students who did and did not complete the modules using independent two-sample t-tests. Of 120 total questions on the medical school exam, 11 questions addressed hepatology topics covered by our gamified modules. Thus, we assessed differences in both total GI exam scores (n=120 items) and hepatology item-specific exam scores (n=11 items), notated as “hepatology exam score” between groups. A p-value <0.05 was considered statistically significant.

Student survey data

Our secondary objective was to obtain qualitative feedback on the modules, including whether gamification increased student interest in hepatology or gamified learning, as well as subjective feedback on efficacy, layout, and technical aspects. Online surveys using Microsoft Forms (Microsoft, Microsoft Forms, Office 365, 2016) were provided to students upon completion of the modules via an embedded link. The survey included 31 questions, with the majority using a five-point Likert scale ranging from strongly agree to strongly disagree (23 questions). The remaining survey question formats included free-form responses (n=4), multiple choice (n=1), true/false (n=1), and two questions asking students to rate their agreement on a Likert scale from one to ten (Appendix 2).

A total of 27 students completed the post-test survey. For analysis, the strongly agree/agree and strongly disagree/disagree categories were collapsed into agree, disagree, or neutral. A result of "agree" in >70% of students defined successful subjective achievement of module objectives.

## Results

Quantitative data: pre vs. post module assessment scores and final exam scores

**Figure 1:**
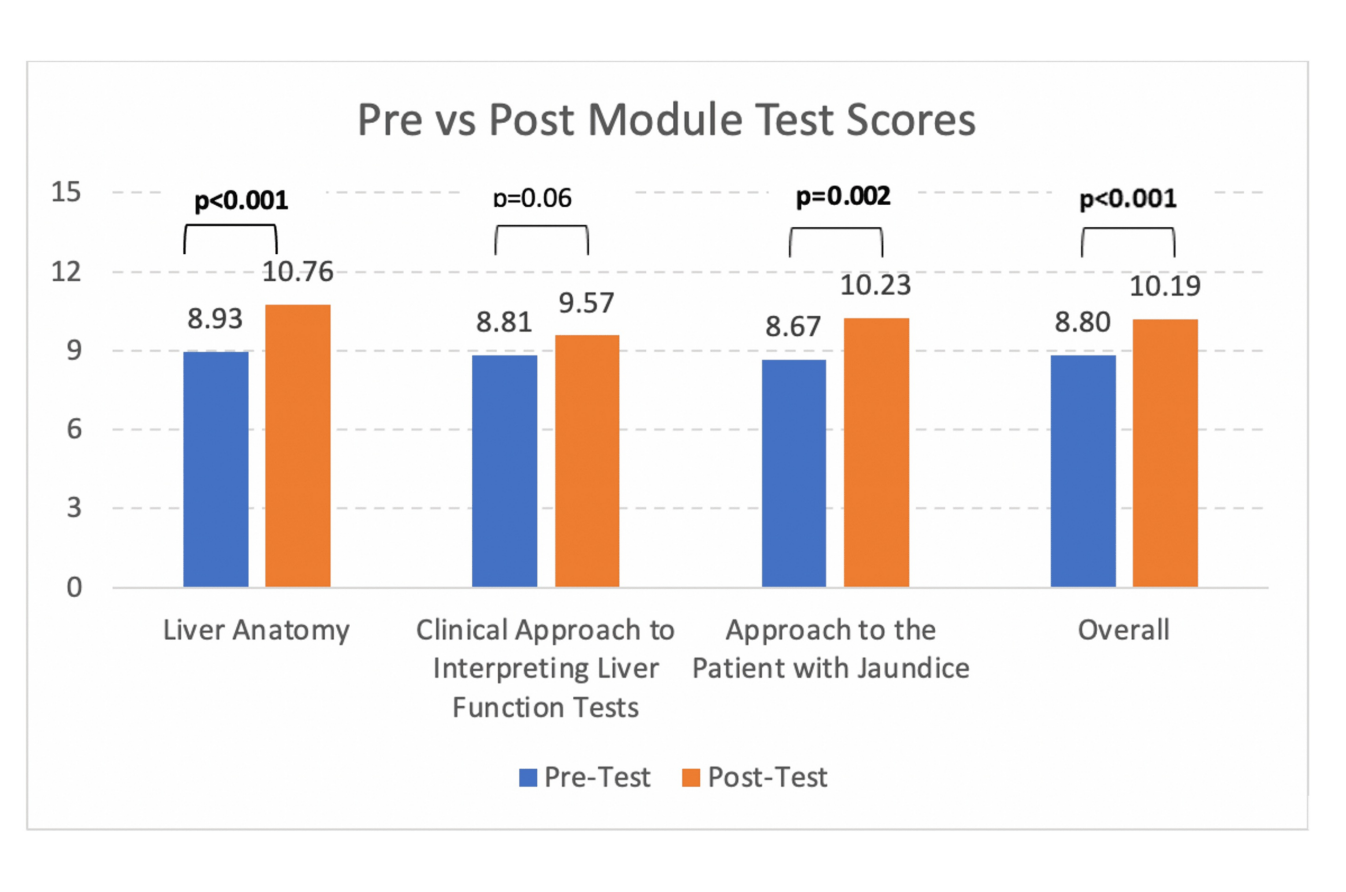
Pre vs. post module test scores of individual modules and all three modules combined.

In total, 13% of students (n=36) completed the modules, and 87% of students (n=240) did not use the modules. A comparison of Medical School GI exam scores between module users and non-module users revealed that module users scored 1.2 points higher on the total GI exam score and 2 points higher on hepatology-specific questions (hepatology exam score). However, there was no significant difference in total exam scores between module users (M=86%, SD=8.67) and non-module users (M=84.8%, SD=8.04); t(274)=(-0.85), p=0.4, Table 1). There was also no difference in hepatology exam scores between module users (M=88.1%, SD=11.25) and non-module users (M=86.1%, SD=10.68; t(274)=(-1.02), p=0.31, Table 1). There was a significant moderately positive correlation between hepatology exam score and total exam score (r=0.57, p<0.0001). Module completion yielded significant increases in pre to post test scores for the Jaundice (Mean pre-test=57% (8.6/15) vs. mean post-test=69% (10.3/15), SD=3.02; t(37)=(-3.42), p=0.002) and anatomy modules (pre-test=61% (9.1/15) vs. post-test=72% (10.8/15), SD=2.57; t(50)=(-4.31), p<0.001), but score improvement was not significantly changed in the LFT module (p=0.06, Table 2, Figure 1). When evaluating a combined module score (aggregate scores of all 3 modules), module completion demonstrated significant improvement in pre vs post test scores (pre-test=59% (8.8/15) vs. post-test=68% (10.2/15), SD=2.82; t(107)=(-5.09), p<0.001, Table 2, Figure 1).

**Table 1:** Descriptive statistics and independent t-test results for hepatology-specific and total exam scores among module and non-module users.

Group	Hepatology Score							Total Score						
	N	Mean Score (%)	SD	Standard Error	T-score	Degrees of Freedom	P-value	N	Mean Score (%)	SD	Standard Error	T-Score	Degrees of Freedom	P-value
Modules	36	88.13	11.25	0.69	-1.02	274	0.3	36	86.04	8.67	0.52	-0.85	274	0.4
No Modules	240	86.17	10.68	1.88				240	84.81	8.04	1.45			
Total	276	86.43	10.76					276	84.97	8.13				
Difference		-1.96	10.76	1.92					-1.24	8.13	1.45			

**Table 2:** T-test paired two sample means of module pre and post test scores. *Two-tailed t-test. LFT: Liver function test.

	N	Mean Pre-Test Score	Mean Post-Test Score	Mean Difference	SD	Standard Error	T-Score	Degrees of Freedom	P-value*
All Modules	36	59%	68%	1.38	2.82	0.27	-5.09	107	<0.001
Jaundice	38	57%	69%	1.68	3.02	0.49	-3.42	37	0.002
LFTs	40	59%	65%	0.86	2.83	0.45	-1.91	39	0.06
Liver Anatomy	51	61%	72%	1.55	2.57	0.36	-4.31	50	<0.001

Qualitative survey data

After completion of the modules, users filled out a survey using Likert scale scoring and free-form responses to obtain qualitative data on students’ subjective perceptions of module efficacy, interest level, and specific feedback on the most and least helpful components. A total of 75% of module users (n=27/36) completed the survey. The survey length was brief, with 31 questions and an average survey response time of 4 minutes and 27 seconds.

Module efficacy

**Figure 2:**
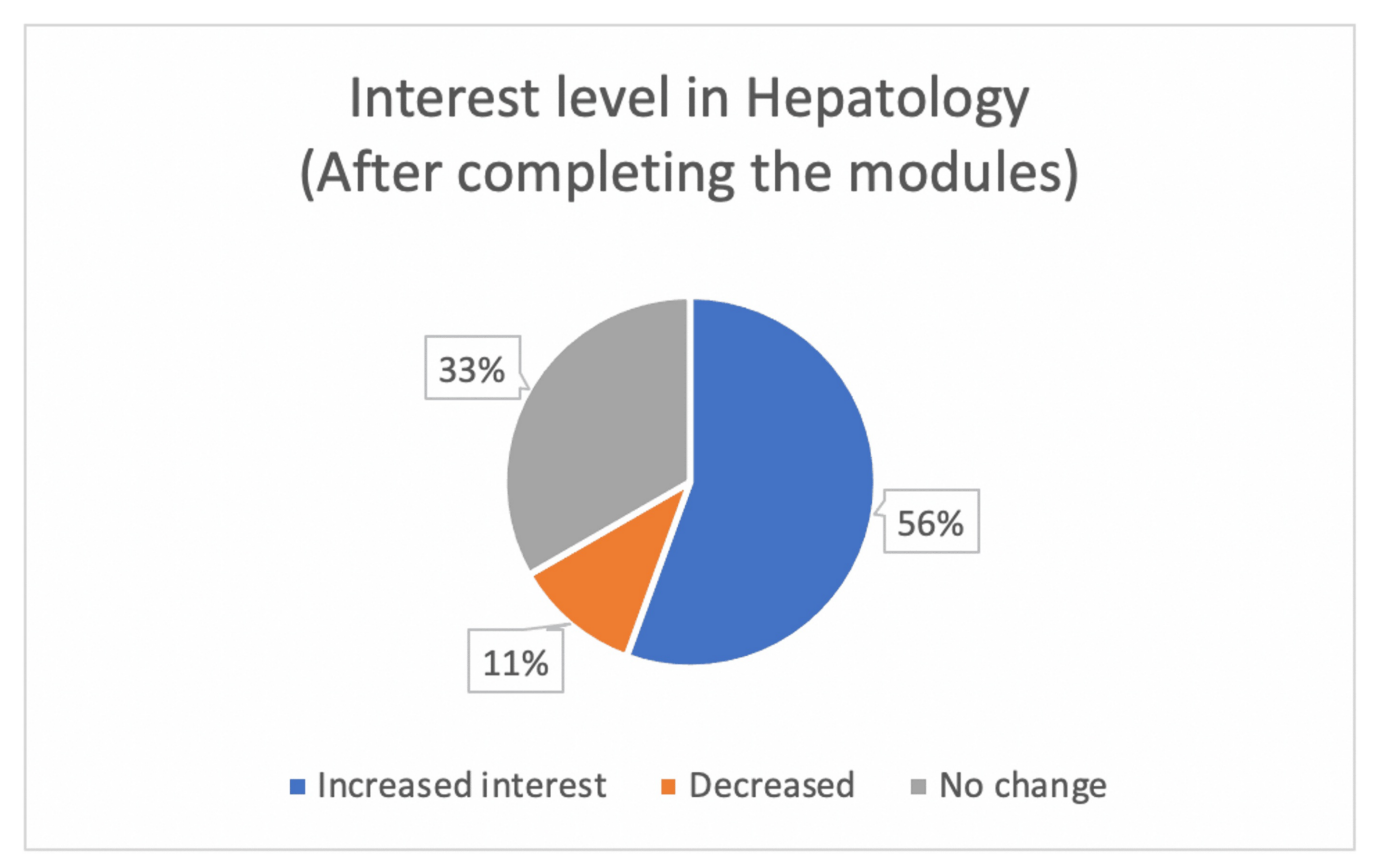
Medical student interest level in hepatology demonstrated in post-module survey.

In terms of effectiveness, the majority of students felt that the modules were interactive (89%), easy to interpret (85%), clear (83%), effective in providing an overview of the content (78%), and aiding in recall (85%). The percentage of students who agreed or strongly agreed that the module accomplished its objectives was highest with the anatomy module (85%) compared to the jaundice (76%) or LFT modules (74%). After reviewing the modules, students felt most confident understanding anatomy concepts such as biliary system structure (96.2%), overall liver anatomy (92.5%), and identifying causes of jaundice (92.6%). On the other hand, scores were lower for applied concepts as only 66.6%, 59.2%, and 66.6% of students felt they could create a differential for elevated LFTs, differentiate when to use magnetic resonance cholangiopancreatography (MRCP) versus ultrasound, or identify basic liver pathology, respectively (Figure 2).

Gamified techniques

A total of 85.1% of students subjectively felt that the quizzes and practice questions improved or strongly improved knowledge recall, while 14.9% of students had a neutral opinion or did not feel the modules enhanced recall. Further, 59% of students felt additional practice questions and games would be beneficial, whereas 11% felt the number of gamified elements were too numerous, and 30% were indifferent. Students were specifically asked to rate the effectiveness of the module mnemonics on a scale from 1 to 10, with 10 being most effective and 1 being least effective. On average, students gave the mnemonics a usefulness score of 6.6/10 (Median=7/10). The most popular student responses regarding their favorite gamified elements included brief knowledge checks (n=7), quizzes/board questions (n=6), animated videos (n=3), interactive cases (n=3), with less popular responses being mnemonics (n=2) and interactive exercises that asked students to draw out anatomy structures or diagram processes (n=1).

Module layout and technical performance

**Figure 3:**
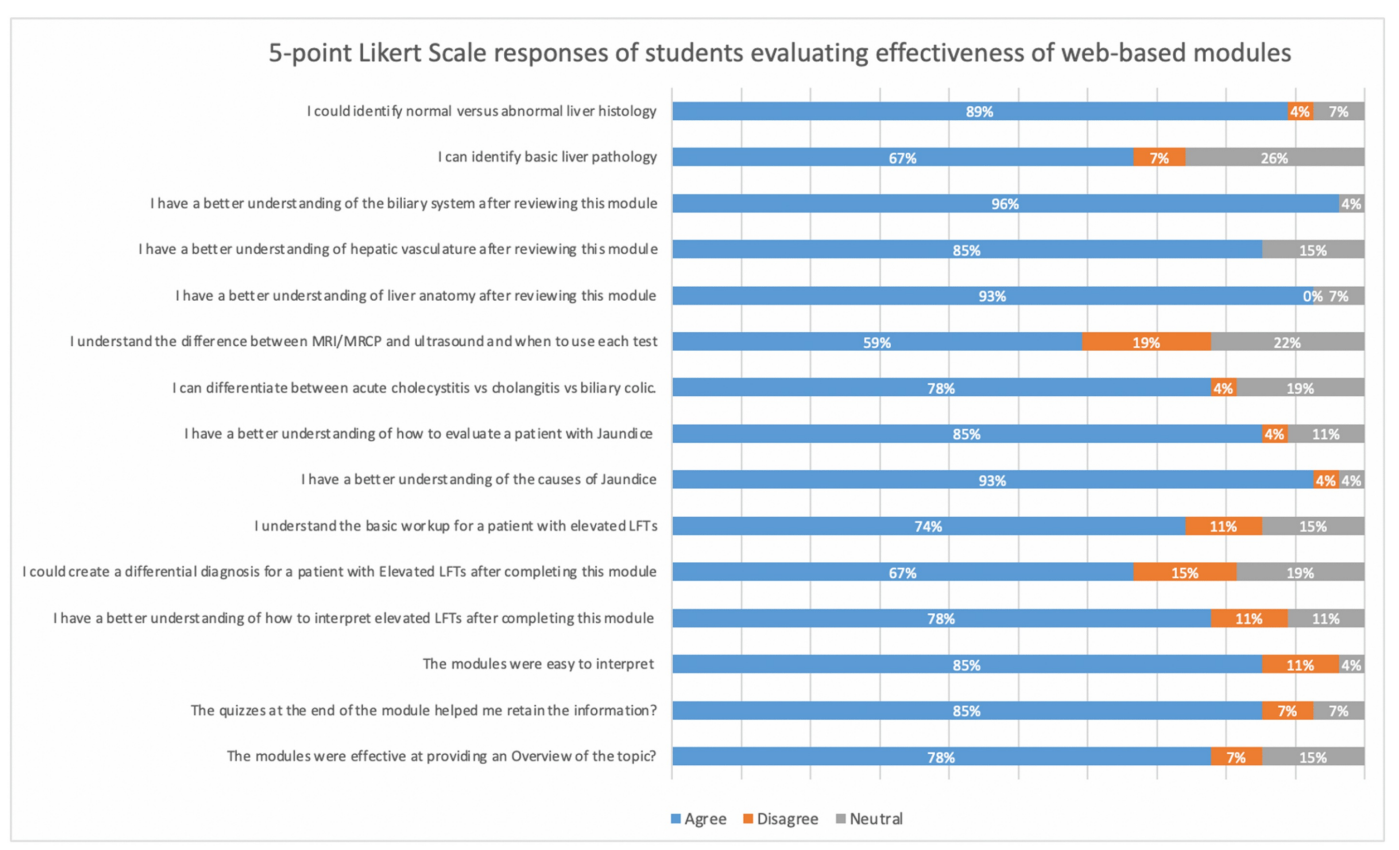
Five-point Likert scale responses of students evaluating effectiveness of web-based modules.

In terms of module layout and technical issues with the online Articulate platform, only 4% of students experienced technical issues, and no students felt the modules were difficult to navigate. Most students found the modules visually stimulating (90%) and interactive (89%). However, 63% of students developed screen fatigue. The majority of students were accustomed to using supplemental web-based materials daily (33.3%) or several times weekly (40.7%), while a minority of students used materials a few times a month (22.2%) or never (3.7%). During their med school hepatology course, students who completed the modules commonly used other supplemental materials (Figure 3).

Impact of modules on student engagement

**Figure 4:**
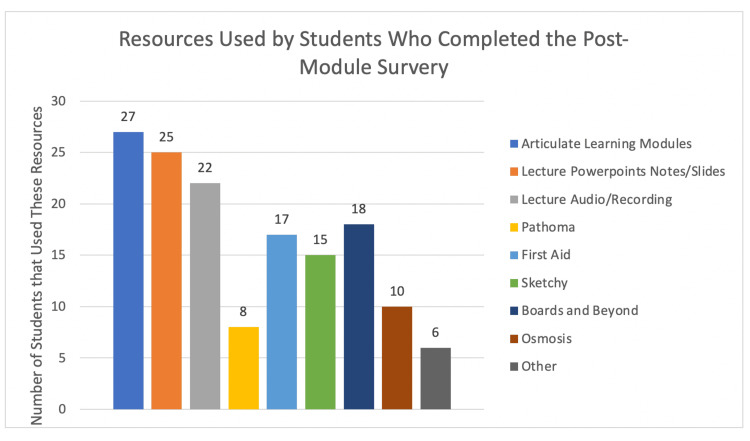
Other medical education resources utilized by medical students.

**Figure 5:**
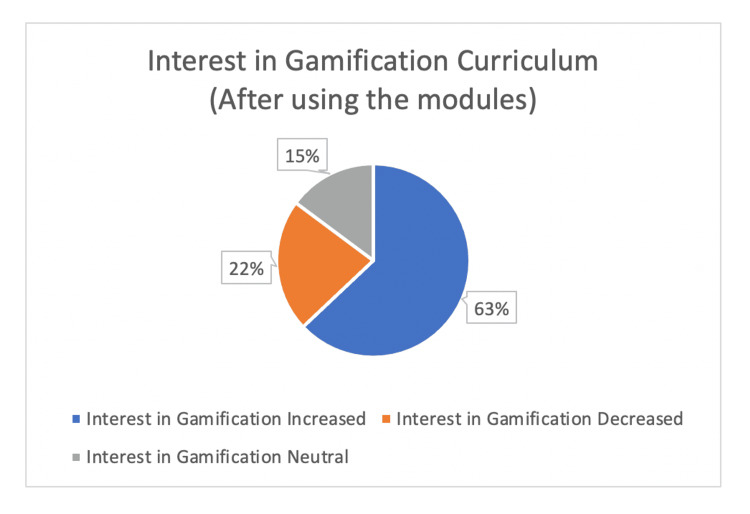
Medical student interest in gamification demonstrated in post-module survey.

At the conclusion of the modules, 63% (n=17) of students had a strong or very strong interest in future gamified curriculums. On the other hand, 22% did not have an increased interest in gamification, and 15% of students had no change in interest level (Figure 4). After completion of the modules, 56% (n=15) of students had an increased interest in hepatology (Figure 5).

## Discussion

There is a paucity of literature examining the quantitative outcomes of gamification in medical education [6]. Our study is the first, to our knowledge, to examine the quantitative and qualitative effects of gamification on a medical school hepatology curriculum. In summary, we found that after the use of the gamified modules, students’ post-test scores were significantly higher on all modules except for the elevated LFT module. Students who used the gamified modules had a non-significant trend towards higher GI block exam scores compared to non-module users. On qualitative surveys, subjective interest in both the gamified curriculum and hepatology increased after module use.

Despite the many advantages of a gamified curriculum, widespread implementation in the field of medical education has lagged behind that of other fields such as industry [6]. Specifically, gamified material can augment content retrieval and enhance learning by forming memories that are more durable in future applications [4]. Gamification often uses Bauman’s layered-learning model, which transforms traditional didactics into a multimodal approach with the goal of supplementing traditional course objectives so that simulation can take place at any time and place [3].

In Bauman’s model, the role of the faculty member shifts from lecturer to a guide who demonstrates how to leverage technology and resources for the benefit of the learner, so they might more actively engage with material and form knowledge connections [7]. The benefits of gamification include increased collaboration, improved clinical decision-making skills, simulation of real-world scenarios, and access to learning analytics [1]. These advantages were mirrored in our study findings of improved module post-exam scores as well as subjective student perception of increased interaction with the material.

Although there is limited data on the quantitative outcomes of a gamified medical curriculum, our results are in line with the existing data. While we noted a significant improvement in module pre to post-exam scores, there was no significant improvement in the GI block exam score among students who used the modules. Interestingly, similar results were noted by Felszeghy S et al. who used the interactive “Kahoot” (Kahoot!: (V.2012), Versvik) platform to create histology games. They noted that the proportion of exam scores was increased in those who used the Kahoot game but not at a statistically significant level [7]. On the other hand, Neureiter’s group found significant improvements in histology quizzes using this same gamified Kahoot platform when comparing pre to post-test scores, in line with our findings [5]. Unfortunately, the majority of current gamification literature remains subjective based on student perception of effectiveness. Thus, dedicated, larger sample studies focusing on quantitative outcomes are essential to gamification's progress.

Our findings suggest that the structure of gamified content may also affect outcomes, and certain techniques may be more effective than others. Specifically, we noted that our approach to the elevated LFTs module was the only one that did not show a significant improvement in module post-test scores. As the LFT content is more theoretical compared to the visual subject matter of the anatomy or jaundice modules, which involved visualizing structures or diagramming pathways, perhaps it was more difficult to engage students with interactive gamified techniques. Through a meta-analysis assessing the pedagogical effect of games on medical education learning, it was found that medical game authors preferred gamifying topics focused on memory and repetition, which are difficult to assess with more conceptual topics and have not been well-studied [8]. Prior research demonstrates that games activate pleasure centers in the brain, leading to dopamine release which can increase student motivation and engagement [9]. McCoy L and Walker J have shown that increased engagement levels subsequently led to higher exam scores [1,4]. Similarly, additional study has shown the converse to be true, with student disinterest revealed to be a key component leading to lower performance in higher education, especially among male students [10]. Ultimately, LFT module length, content nature, and perhaps student interest levels could have impacted the lower scores.

Consistent with existing literature, our survey revealed that students perceived that their use of the gamified modules contributed to recall and learning effectiveness. Gue S et al. applied gamification to medical residency education and noted similar results to our survey of increased motivation, engagement, and perceived enhanced recall versus traditional didactics [11]. Education theory asserts that scaffolding occurs more effectively in the setting of discussion and activities rather than passive lecture. As games are a type of collaborative discussion, this may explain the increased recall and outcomes noted with gamification in our study as well as others [1,12,13]. Gamification in medical education provides exciting avenues that explore collaboration, as well as competition, which notably improves motivation and engagement in learning [14].

## Conclusions

In regards to next steps, we aim to objectively explore which specific game elements are most effective in enhancing student performance. We plan on making adjustments to our modules based on survey feedback with respect to module length and incorporation of favored student game elements. We also aim to augment the four components of serious games, including making sure players are challenged with a purpose and continued orientation, rules that guide players' growth and achievement, continuous feedback to guide players toward success, all of which occur through voluntary participation. Our study has several strengths, including its prospective design and the use of an experienced, diverse team to design our modules, which includes medical, educational, and design professionals such as an education curriculum consultant and a graphic designer. We also used two separate quantitative measures to judge effectiveness: both GI course exam scores as well as gamified module pre and post-test scores. However, our study was limited by its single-center design and small sample size. The lack of difference in GI block exam scores may be due to a type 2 error, as module pre and post-test scores were significantly improved overall and in all modules except the LFT module. Another obstacle that we face in showing a significant difference in GI block exam test scores is the sheer number of resources that medical students utilize on a regular basis. With multiple methods to learn the same information, it is difficult to show that our gamification modules alone are able to effect significant increases in their GI block exam scores. Finally, baseline demographic data for students was not available. Thus, we cannot rule out that an inherent common characteristic in the module group could have biased their performance.

Overall, our study showed that an online, gamified hepatology curriculum was effective as evidenced by significant improvements in pre to post-module exam scores and was associated with a trend towards higher average exam scores among highly engaged users. Through a qualitative post-module survey, most students perceived the modules as beneficial to retaining hepatology knowledge and had increased interest in using web-based interactive formats in the future. Larger sample studies with investigation of student characteristics are needed to further define the role of a gamified hepatology curriculum. While our discussion emphasizes medical student engagement pre-clerkship, future studies should assess whether gamification continues to pique interest in hepatology during clerkships and if there is higher retention of hepatology material among module users when they are in the clinical setting.

## Appendices

Appendix 1

Module Links

**Table 3:** Links to hepatology modules.

Name of Module	Liver Anatomy	Approach to the Patient with Jaundice	Clinical Approach to Interpreting Liver Function Tests
Direct Link	https://rise.articulate.com/share/IoJa1llOeNnymjmoNbiuBofMl2HoqrDJ#/	https://rise.articulate.com/share/6gQZjEek_M6ZoOgf2lUhiKa6oZ-9ap5G#/	https://rise.articulate.com/share/RYtdaPjXKSqMfr-TgNFDYP5fibclcAkU#/

Appendix 2

Post-Module Survey Content

**Table 4:** Assessing all resources used for exam.

Survey question: Please select which resources you used to study for this exam:
Articulate Learning Modules (study’s modules)
Lecture Powerpoints from class
Lecture audio/video from class
Pathoma
First Aid
UWorld
Sketchy Micro/Pharm
Boards and Beyond
Other (Please list)

**Table 5:** Assessing module content/effectiveness questions. *Questions answered with Likert Scale: 1: Very effective; 2: Effective; 3: Neither effective nor ineffective; 4: Ineffective; 5: Very ineffective. **Questions answered by following numerical scale: 1: Strongly agree; 2: Agree; 3: Neither Agree nor Disagree; 4: Disagree; 5: Strongly Disagree. ***Questions answered by following numerical scale: 1: I could definitely do this; 2: I could mostly do this; 3: I could do some of this; 4: I would struggle with this; 5: I could not do this.

Assessing Module Content/Effectiveness Questions
Using the 5 point Likert scale below, please rate how effective the curriculum was at giving you an overview of the topic?*
Did you feel the workbook activities at the ends of the modules helped to retain information?*
I have a better understanding of how to interpret elevated LFTs after completing this module**
I have a better understanding of the causes of jaundice after completing this module**
I have a better understanding of liver anatomy after reviewing this module**
I have a better understanding of hepatic vasculature after reviewing this module**
I have a better understanding of the biliary system after reviewing this module**
The images describing the anatomy were easy to interpret**
I could identify basic liver pathology***
I could identify normal versus abnormal liver histology ***

**Table 6:** Assessing module layout/overall student interest questions. *Questions answered by following numerical scale: 1: Strongly agree; 2: Agree; 3: Neither Agree nor Disagree; 4: Disagree; 5: Strongly Disagree.

Assessing Module Layout/Overall Student Interest Questions
The modules have increased my interest in hepatology*
This experience has increased my interest in using a web based or interactive format, compared to traditional lecture style*
I thought the modules were visually stimulating*
I found the modules to be interactive*
I would have preferred more quizzes/games*
Please rate how clear the content was on a scale of 1-10?
How helpful were the mnemonics on a scale of 1-10?
What features of the modules did you like best?
What did you like least?
What was confusing to you?
What feature would you like to see more of?

**Table 7:** Assessing module technical aspects. *Questions answered by True/False **Questions answered by following numerical scale: 1: Very difficult; 2: Somewhat difficult; 3: Neither difficult nor easy; 4: easy; 5: Very easy. ***Questions answered by following numerical scale: 1: Strongly agree; 2: Agree; 3: Neither Agree nor Disagree; 4: Disagree; 5: Strongly Disagree.

Assessing Module Technical Aspects
I did not experience technical issues *
Describe how difficult or easy the module was difficult to navigate**
I experienced screen fatigue while using the modules***
